# Family Size and Parental Wealth: The Role of Family Transfers in Europe

**DOI:** 10.1007/s10680-022-09611-w

**Published:** 2022-03-31

**Authors:** Zachary Van Winkle, Christiaan Monden

**Affiliations:** 1 Sciences Po, Observatoire Sociologique du Changement (OSC), CNRS, 27 Rue Saint-Guillaume, Paris Cedex 07, 75337 Paris, France; 2 Department of Sociology and Nuffield College, University of Oxford, Oxford, United Kingdom; 3 Leverhulme Centre for Demographic Science, Oxford, United Kingdom

**Keywords:** Wealth, Family size, Inequality, Childless, Family, Comparative, Family transfers

## Abstract

As baby boomers enter retirement, an increasing portion of the population in Europe will rely on wealth as a source of financial security. We address two research questions: what is the association between family size, i.e. the number of children, and wealth for adults who are preparing for or have entered retirement and does the generosity of family transfers moderate that association? Data from the Survey of Health, Ageing, and Retirement in Europe (SHARE) are used to estimate the relationship between family size and the total household net worth of men and women between ages 50–65, born 1939–1967 from 14 European countries. We use logistic and linear regression modelling to investigate the probability of zero or negative wealth and net worth percentile rank. We find that adults with four or more children are more likely to be in debt and have less wealth than childless adults. In contrast, adults with two and three children have more wealth. We provide evidence that the generosity of family transfers ameliorates the negative association between larger family sizes and wealth, but may exacerbate wealth inequality by benefiting two and three child families most.

## Introduction

Interest in wealth as a crucial dimension of social inequality has increased dramatically in the last two decades among both scholars and the general public (Killewald et al., 2017; Piketty, 2014). While sociological research has focused on wealth’s role in the intergenerational transmission of social status (Keister & Moller, 2000; Spilerman, 2000), economists have concentrated on how individuals accumulate wealth through spending and saving (Deaton, 2005; Modigiani & Brumberg, 1954) and whether those patterns vary between households with and without children (Modigliani, 1986). The findings of recent sociological and economic studies on the relationship between the number of children and wealth are nonetheless mixed (Scholz & Seshadri, 2007; Tin, 2000; Yamokoski & Keister, 2006; Schmidt & Sevak, 2006; Lersch et al., 2017; Dockery & Bawa, 2015a, 2015b). However, the relationship between family size and accumulated wealth can have serious consequences for society, especially at a time when pension systems are under pressure from demographic change.

Despite the increased interest in wealth accumulation among households of various sizes, there are a number of critical gaps in the literature. First, most sociological and economic research on the association between family size and household wealth conducted on non-US data is limited to Germany, the UK and Australia (Dockery & Bawa, 2015a, 2015b; Lersch & Dewilde, 2018; Lersch et al., 2017) with few comparative designs (see Semyonov & Lewin-Epstein, 2013 for an exception). This has important implications for our understanding of how family size impacts the amount of wealth that individuals accumulate. Due to the lack of cross-national studies on the relationship between family size and wealth, theoretical considerations about how and to what extent family transfers can moderate the association between family size and wealth remain underdeveloped and empirically untested. Second, most studies focus on how the number of children or the transition to parenthood affects wealth accumulation while adults are still relatively young. However, it is important to observe wealth differences for older adults with and without children. Couples may save disproportionately after children leave the household to prepare for retirement, which could lead to biased estimates. Further, wealth has a greater meaning for older adults, as it becomes one of their primary source of income and financial security.

In this study, we address these gaps with two research questions: First, what is the association between family size, i.e. the number of children, and household wealth for adults who are preparing for or have entered retirement? Specifically, we test competing assertions on how the presence of children influence wealth accumulation over the life course. On the one hand, parents may save or invest more of their disposable income and accumulate more wealth than childless adults with the intention of leaving their children an inheritance. On the other hand, the costs of children may hinder wealth accumulation and leave parents with less wealth than childless adults. Second, does the generosity of family transfers, i.e. the extent that countries compensate families for the costs of children, moderate the association between family size and wealth?

We use data from the Survey of Health, Ageing, and Retirement in Europe (SHARE) to estimate the relationship between family size and the total household net worth of men and women between ages 50–65, born 1939–1963 from 14 European countries. In addition, we draw on Gauthier's (2011) comparative family policy dataset to estimate whether the generosity of family transfers moderates the association between family size and wealth. We use logistic regression modelling to investigate the probability of zero or negative wealth and linear regressions to assess family size differences in net worth percentile rank across contexts with varying generosity in family transfers.

This study contributes both theoretically and empirically to the literature on wealth accumulation and inequality. More specifically, we demonstrate that whether the number of children is associated with wealth depends on two factors that have been overlooked in past research. First, previous mixed findings on the relationship between family size and wealth may be attributable to measuring the number of children continuously. While we find adults with four or more children are more likely to be in debt and own less wealth than childless adults, two and three children families are in an advantaged position with a lower probability of zero or negative wealth and more wealth than childless adults. Second, previous studies have not accounted for contextual differences. We extend the life-cycle models of wealth accumulation found in both the sociological and economic literatures by accounting for the generosity of family transfers when hypothesizing about how the number of children will influence wealth accumulation. Indeed, we provide evidence that the generosity of family transfers ameliorates the negative association between larger family sizes and wealth, but strengthens the positive association between two and three child families and wealth.

## Previous Research on Family Size & Wealth

To date, most of the studies on family size and wealth, or studies that report results on family size and wealth, have been performed on US data. Nearly two decades ago, Keister and Moller (2000) concluded in their review of wealth inequality in the US that family size likely decreases wealth ownership (e.g. Land & Russell, 1996; Maroto, 2017; Tin, 2000). For example, using data from the US Health and Retirement Study (HRS), Scholz and Seshadri (2007) demonstrate that the number of children decreases wealth by reducing resources available for consumption or saving. Further, they argue that after variation in family size has been accounted for, means-tested cash and near-cash transfer programs have little impact on household wealth.

However, a number of studies have reported positive associations between family size and wealth (e.g. Bernardi et al., 2019; Bogan, 2013; Yamokoski & Keister, 2006). Grinstein-Weiss and colleagues (2008), for example, used the 2001 Survey of Income and Program Participation (SIPP) to show that married households with three or more children have higher net wealth than childless households. Other studies have found no association between family size and wealth (e.g. Ozawa & Lee, 2006; Painter et al., 2015). Tamborini and Purcell (2016) use the 2001–2010 Survey of Consumer Finances and find that the number of children in the household is not associated with coupled women’s retirement account wealth.

Some studies highlight that the relationship between family size and wealth may be simultaneously positive and negative, thus leading to mixed or null findings (e.g. Smith & Ward, 1980). Schmidt and Sevak (2006) use the Panel Study of Income Dynamics to demonstrate that, on average, having older children is negatively related to household wealth. However, they present some evidence that having children may be positively associated with wealth for households above the 50th percentile in the conditional wealth distribution. Recently using the 1979 National Longitudinal Survey of Youth NLSY, Maroto (2018) finds that the association between parenthood and net wealth varies starkly across the unconditional wealth distribution: parenthood is negligibly associated with wealth below the 15th percentile, then is associated with an up to 40 percent decrease in wealth between the 20th and 50th percentile before the association becomes positive. Among the wealthiest families, parenthood increases net total wealth by well over 100 percent.

The few studies that report on the association between family size and wealth on non-US data are also mixed. Semyonov and Lewin-Epstein (2013) use data from 13 countries from SHARE, HRS, and the English Longitudinal Study of Ageing, to estimate the associations between a number of factors and wealth. They report that household size is not associated with net wealth in a pooled sample of 16 countries, but they do find negative associations in France and the United Kingdom as well as positive associations in Denmark, Switzerland, Belgium, and the USA. More recently using data from the British Household Panel Survey (BHPS) and Socio-Economic Panel Study (SOEP), Lersch and Dewilde (2018) find that underage children in the household decrease the amount of money saved each month in both the UK and Germany (see also Lersch et al., 2017 for Germany; Dockery & Bawa, 2015a, 2015b for Australia).

Studies do not only vary according to the data they are based on, but also how the association between family size and wealth is modelled. However, these methodological differences do not seem to systematically correspond with a specific conclusion. For example, previous research using single or pooled cross-sectional data with OLS, probit, or tobit regression models have found positive (Bogan, 2013; Grinstein-Weiss et al., 2008), negative (Land & Russell, 1996; Tin, 2000), mixed (Smith & Ward, 1980), and null results (Ozawa & Lee, 2006; Tamborini & Purcell, 2016). Studies based on longitudinal data that do not observe fertility data, such as the current study, commonly employ pooled OLS regressions or growth curve modelling and have found also found positive (Bernardi et al., 2019), negative (Scholz & Seshadri, 2007), as well as mixed and null findings (Semyonov & Lewin-Epstein, 2013). Even those studies that might be considered that golden standard of employing longitudinal data that include fertility transitions with panel models have found positive (Yamokoski & Keister, 2006), negative (Lersch et al., 2017; Maroto, 2017), mixed (Maroto, 2018; Schmidt & Sevak, 2006; Lersch & Dewilde, 2018), and null results (Painter et al., 2015).

The majority of these studies either compare parents with childless adults (Tin, 2000; Maroto, 2018; Schmidt & Sevak, 2006; Lersch et al., 2017, 2018) or model a linear association between the number of children and wealth (Bogan, 2013; Dockery & Bawa, 2015a, 2015b; Land & Russell, 1996; Painter et al., 2015; Semyonov & Lewin-Epstein, 2013; Smith & Ward, 1980; Tamborini & Purcell, 2016; Yamokoski & Keister, 2006). Few studies use a categorical indicator of family size to allow nonlinear associations between the number of children and wealth (Grinstein-Weiss et al., 2008; Ozawa & Lee, 2006).

In sum, a review of the literature shows that more research is needed to understand the relationship between family size and the number of children. Foremost for our purposes, context matters: there are stark contrasts between results from different countries. Killewald et al. (2017) in their review of wealth inequality and accumulation conclude that there is still a lack of cross-national research and that little is known on the specific institutional and economic determinants of wealth inequality.

## Theoretical Background

### Family Size and Wealth

**Fig. 1:**
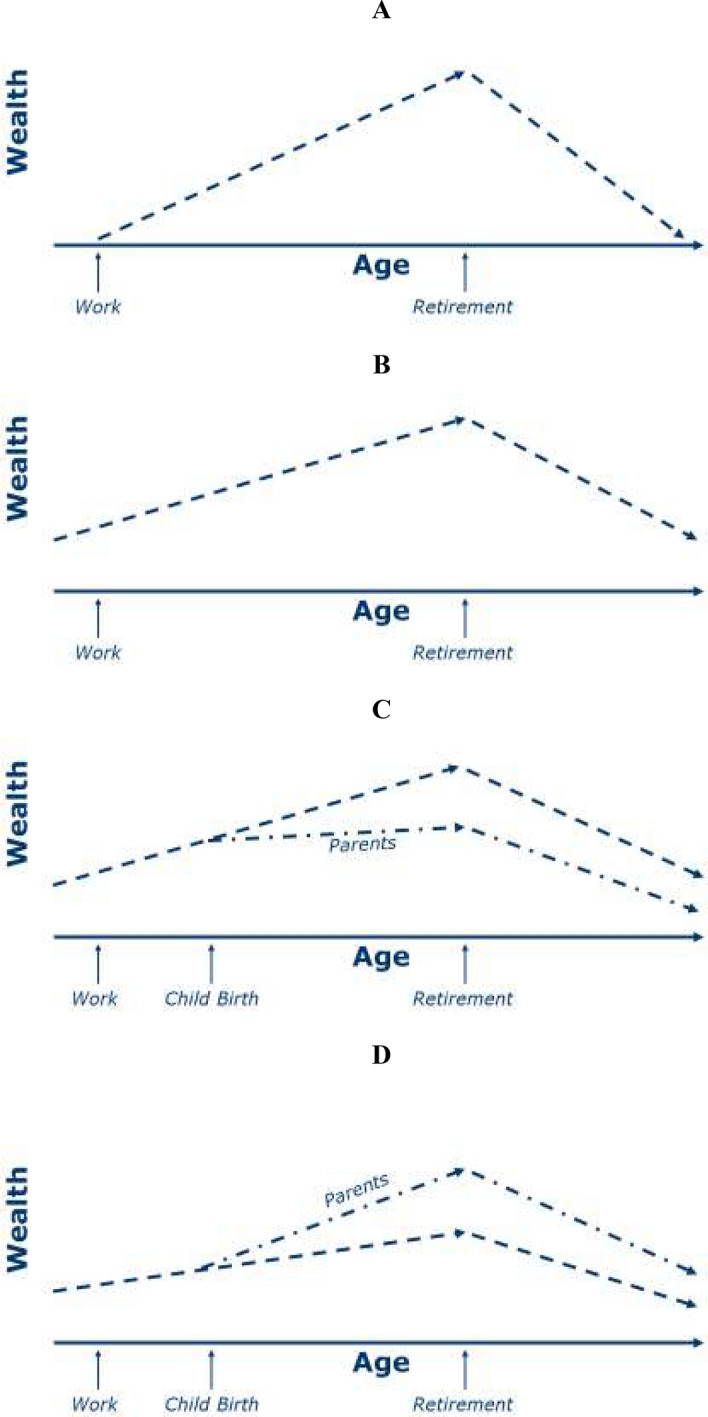
The Life-Cycle Hypothesis for Wealth Accumulation with and without Children

Why should we expect wealth differences between individuals and households with smaller and larger families? The traditional life-cycle hypothesis initially developed by Modigiliani and Brumberg (1954) conceptualizes wealth accumulation in terms of a save and spend model (Deaton, 2005). The model assumes that disposable income can either be consumed, i.e. spent, or saved. Rational actors will save their income that is not spent while active on the labour market, thereby accumulating wealth. Following retirement, the accumulated wealth will be spent in total. This model is displayed in panel A of Fig. 1. A first revision of the life-cycle hypothesis incorporates the observation that wealth is not saved and spent within one generation, but is inherited from the former generation and bequeathed to the next, as is displayed in panel B of Fig. 1.

However, even the revised life-cycle model proved too static for an adequate representation of how wealth is accumulated during different life course stages or among different segments of the population. Saving and spending patterns vary with the amount of disposable income and household needs, which influence the rate at which wealth is accumulated or consumed (Modigliani, 1986).

The transition to parenthood and the number of children in the household are among the main factors that affect disposable incomes. Motherhood earnings penalties, often estimated as the negative change in earnings as women enter parenthood, are well documented (e.g. Budig & Hodges, 2010). Explanations for such penalties include selection, e.g. more or less productive and work oriented individuals might select into parenthood, lower productivity due to the loss of human capital or a limited ability to fulfil the ideal worker norm of reliability, flexibility and working long hours (Weeden et al., 2016), and discrimination against mothers in terms of hiring, firing, wages, and promotions (Correll et al., 2007). Regardless of why or how motherhood penalties are generated, they suggest that the disposable incomes of households will be negatively affected by women’s transition into motherhood. Although the reduction in household income may be compensated by fatherhood premiums, i.e. often estimated as the positive change earnings as men enter parenthood, research suggests that these are likely smaller in size than motherhood penalties (Killewald, 2013; Van Winkle & Fasang, 2020) or that there are no premiums (Kunze, 2020; Mari, 2019).

The transition to parenthood and the number of children in the household also affect household needs through both the direct and indirect costs of children. Direct costs include all the additional costs that are incurred by households with a dependent child, e.g. food, clothing, childcare, housing, etc. Indirect costs include the loss of income in both the short and long term that are incurred as a result of the presence of children, e.g. mothers’ employment reductions, wage penalties, loss of pension rights, etc. In a report to the European Union on the costs of children, Letablier and colleagues (2009) estimate the relative direct cost of a first child to be between 20 and 30 percent the budget of an average childless couple.

If household incomes decrease following childbirth and household needs increase, then it follows that households have fewer resources to consume or save. In particular, if children increase household needs and reduce parents’ ability to save, as is displayed in panel C of Fig. 1, then we would expect *the number of children to be associated with a higher probability of zero or negative wealth and less total net worth after age 50 (H1a)*. Another possibility however, is that couples save more following childbirth to prepare for the costs associated with childrearing and bequest motives (Land, 1996). That is, even though parents have less disposable income to consume or save, they save a larger absolute and relative portion of that income than childless households. In this case, as displayed in panel D of Fig. 1, we would expect *the number of children to be associated with a lower probability of negative or zero wealth and higher total net worth after age 50 (H1b)*.

The majority of studies reviewed above have either assessed how childless adults and parents differ in wealth or how the number of children is linearly associated with wealth. However, the association between children and wealth may be neither dichotomous nor continuous, but nonlinear (Grinstein-Weiss et al., 2008). For example, although the absolute household costs of children continue to increase with each additional child, the marginal cost of each additional child decreases due to economies of scale (Letablier et al., 2009). Therefore, it could be that only or especially the first child has a negative impact on wealth.

In contrast, the so-called second child syndrome highlights the emergence of gender inequalities and a traditionalizaion of couple relationships following birth of the second child (see Doren, 2019). One the one hand, it could be that the second child—rather than the first—has a particularly negative impact on wealth if mothers withdraw from the labour market after a second childbirth. On the other hand, the two child ideal is one of the most persistent family norms in Europe (Goldstein et al., 2003; Sobotka & Beaujouan, 2014) and may be coupled with higher wealth levels (Lawson & Mace, 2010). For example, fulfilling the second child norm could invoke parents to concentrate more resources on building wealth to be able to support both children and fulfil bequest norms later in life. In sum, an alternative hypothesis to H1a and H1b is that *one and three or more children are associated with a higher probability of negative or zero wealth and less total net worth after age 50, while two children are associated with a lower probability of negative or zero wealth and higher total net worth after age 50 (H1c)*.

### The Role of Family Transfers for Family Size and Wealth

While most European countries provided little public family support before the onset of the Second World War, state provisions for families increased substantially during the post-war period (Gauthier, 1999; Van Winkle, 2019). In this study, we concentrate on one of the most common 20th Century familistic measures in Europe: generous family allowances and other monetary transfers to households with children (Leitner, 2003; Saraceno, 2016). The aim of these measures was to incentivize marriage and parenthood and to support a male-breadwinner female-homemaker division of labour. In the light of decreasing fertility rates across much of Europe (Billari, 2008), the aim of these measures was, at least implicitly, pro-natalistic. The assumption is that family demographic processes and events, such as parenthood, are rational decisions and the result of a utility maximization process (Becker, 1960; see Gauthier, 2007 for a critical discussion). Generous family transfers, the focus of this article, should reduce the economic (opportunity) costs and/or benefits of entering parenthood and having additional children. However, most evidence suggests that the effects of these measures on fertility are small or even negligible (Balbo et al., 2013). What has not been studied is whether or to what extent the generosity of family transfers impact the wealth accumulation of parents.

If family transfers targeted directly or indirectly at meeting families’ consumption expenditures reduce the monetary costs of children, then households with children have more disposable income to consume or save. Therefore, we expect that *the negative association between family size with the probability of negative or zero wealth and total net worth is smaller in contexts with more generous family transfers (H2a)*. In contrast, family transfers may give households with children that save more than childless households do an additional wealth advantage. In other words, we expect that *the positive association between family size with the probability of negative or zero wealth and total net worth is larger in contexts with more generous family transfers (H2b)*. Alternatively, family transfer schemes may be designed to benefit and support families meeting the two child ideal to a greater extent than families with a single child or families with many children, albeit while continuing to mitigate negative wealth consequences of smaller and larger families. Specifically, it could be expected that *the negative association between one and three or more children with the probability of negative or zero wealth and total net worth is smaller in contexts with more generous family transfers, while the positive association between two children with the probability of negative or zero wealth and total net worth is larger in contexts with more generous family transfers (H2c)*.

## Data & Methods

### Sample

To test our hypotheses, we draw on waves 1, 2, 4, 5, 6, and 7 of SHARE (Börsch-Supan et al., 2013). SHARE is a household panel study fielded on a biennial basis that collects a wide range of economic, social, demographic, and health data on respondents aged 50 or older and their partners residing in a number of European countries and Israel. The first wave was collected in 2004 and 2005 in 11 countries and in 2005 and 2006 in Israel. The second wave was collected in 2006 and 2007, and included three new countries. Note that we do not use observations from the third wave of SHARE, also referred to as SHARELIFE, and SHARELIFE observations in the most recent wave, because the life history modules did not collect a number of the variables used in our analyses. The fourth, fifth, sixth, and seventh waves were collected in 2011, 2013, 2015, and 2017, respectively, with minor country differences in timing.

We restrict our sample to respondents and their partners aged 50–65, because we are interested in wealth accumulation leading up to retirement. Therefore our oldest respondents were born in 1939, i.e. age 65 in 2004, and our youngest were born in 1967, i.e. age 50 in 2017. This restriction additionally reduces mortality bias in our analyses. To increase comparability across households, we include only single and couple households and exclude respondents living in nursing homes. As will be discussed further below, high quality data on the generosity of family transfers is only available for 14 Western European counties. Therefore, we can only include individuals residing in Austria, Belgium, Switzerland, Germany, Denmark, Spain, France, Italy, the Netherlands, Sweden, Ireland, Luxemburg, Greece, and Portugal. Note that our sample pools all observations, i.e. person-years, and includes respondents that were observed at least once.

### Dependent Variables

We measure wealth as the total net worth of the household, which encompasses both household real assets and household net financial assets. Household real assets is the sum of the proportional value of the primary residence owned by the respondent, the proportional value of the respondent’s business owned by the respondent, the values of automobiles and other real estate, minus the respondent’s mortgage on the main residence. Household net financial assets is the sum of bank accounts, bonds, stock and mutual funds, and savings for long-term investments, minus financial liabilities. We convert household net worth to purchasing power parities equivalent to 2015 Euros in Germany.

The frequency of missing values among wealth variables in SHARE is high. We therefore draw upon the imputed data that SHARE provides for observations that were not the designated household respondent, i.e. information provided by another person in the household, or imputations based on unfolding brackets range information. In the latter case, respondents that did not provide information for wealth variables were presented with a card containing three country-specific values and asked whether their own value (a) lies below the lower value, (b) around the lower value, (c) between the lower and mid-value, (d) around the mid-value, (e) between the mid- and upper value, (f) around the upper value, or (g) above the upper value. We do not use values that are imputed based completely on other information and hot-deck imputation methods. When using values from the designated respondents and imputed values based on unfolding brackets range information, the number of missing values is considerably reduced.

To test our hypotheses, we analyse two wealth variables: zero or negative wealth and net worth percentile rank. Zero or negative wealth is a binary variable that takes the value of zero in the case of positive total net worth and takes the value of one in the case of negative or zero total net worth. Net worth percentile rank, which includes negative and zero wealth, is analysed as percentiles of total net worth. We chose this transformation, because it reduces the skew of the wealth distribution and the influence of outliers while allowing us to include the full range of wealth, including both negative and very large positive values (Killewald et al., 2017). In addition, the interpretation of results based on wealth percentiles is more intuitive than results based on the commonly used inverse hyperbolic sine, which also reduces the skew of the wealth distribution while including negative values. Results based on the inverse hyperbolic sine of wealth are substantively similar to those presented below.

### Independent Variables

Family size is measured as the number of biological or adoptive children of both the respondent and their spouse. We include family size as a categorical variable (childless, one child, two children, three children, or four plus children) to account for nonlinear associations between family size and wealth as postulated by our hypotheses H1c and H2c.

We use Gauthier’s (2011) comparative family policy database to create a country-cohort indicator on the generosity of family transfers. For all our study countries, we have an annual indicator for the total tax and benefit transfers for a two-parent, two-child, one-earner family expressed as the percent of average gross earnings of a production worker. We then calculate country-cohort specific values by averaging over the years that individuals were between age 20 and 45. For example, we average the benefit values from 1980 to 2005 for individuals born in 1960. Therefore our index expresses the generosity of family transfers experienced by individuals over their life course, rather than at a single point in time (see Van Winkle 2019; Van Winkle & Fasang, 2017). Note that we do not have complete information for individuals born before 1952, but use all the information available when creating their averages.

### Analytical Strategy

We use two sets of regression models to estimate the association between family size and wealth as well as the interaction between family size and the generosity of family transfers. First, we model the associations between family size, the generosity of family transfers, and the probability of zero or negative wealth using logistic regression models. Second, we use OLS linear regressions to estimate the associations between family size, the generosity of family transfers, and the net worth percentile rank. As we pool multiple observations across waves, i.e. multiple person-years, we estimate robust standard errors that are clustered by persons. Standard errors clustered by countries to address potential heteroscedasticity due cross-national sampling were generally smaller for our estimates of interest, which is why we display the more conservative standard errors clustered by persons below (see Table 6).

For the logistic regressions on zero or negative wealth and linear regressions on net worth percentile rank, we estimate three models each. The first set of models estimate the association between family size and zero or negative wealth or net worth percentile rank, which include only country and birth year fixed effects as well as age, age-squared, and gender. The inclusion of country and birth year fixed effects will adjust for all time-constant country differences and any birth year differences that are shared across countries. The second set of models adjust for factors that are associated with selection into parenthood and with zero or negative wealth or net worth percentile rank. However, we are careful to only include variables whose omission can lead to confounding bias and do not include mediators. We include three candidate confounders: respondents’ educational attainment (in years as a quadratic term), whether the respondent was ever married, and the number of rooms in the childhood home. Although some respondents may have entered parenthood before completing education or entering marriage, it is likely a negligible proportion for the birth cohorts in our sample. We use the number of rooms in the respondents’ childhood home as a proxy for their socio-economic background. Alternative measures, such as parental education, may well serve as better proxies for socio-economic background, but parental education has was only collected in the last three waves of SHARE. The number of rooms in the parental household was additionally collected in the third wave of SHARE. The final set of models include an interaction between our family transfers indicator and family size. All models are weighted to correct for sampling probabilities and panel attrition.

After listwise deletion, our analysis sample consists of 75,228 person-years nested within 39,177 persons. The majority of cases not included in the analyses are due to missing wealth information (28,915) although a small number are excluded due to missingness on other variables included in the models, such as educational attainment (3082). A comparison of the socio-demographic composition of all cases without exclusions, those with missings on wealth, those with missings on other variables, and our final analysis sample show only minor differences (see Table 7). For example, those with missing wealth information are slightly more educated (12.2 years with missing wealth information compared to 11.5 years in the sample prior to listwise deletion and in the 11.3 years in the analysis sample) and more likely to be women (53 percent with missing wealth information compared to 51 percent in the sample prior to listwise deletion and in the 50 percent in the analysis sample). However, these small differences are unlikely to grossly bias our estimates.

## Results

### Descriptive Statistics

The proportion of observations with negative or zero total household net worth are displayed in Table 1 for western European countries (Austria, Belgium, Switzerland, Germany, France, the Netherlands, Ireland, and Luxemburg), Scandinavian countries (Sweden and Denmark), and southern European countries (Spain, Italy, and Portugal). Family size differences in zero or negative wealth are considerable. Childless adults have high proportions of zero or negative wealth, ranging from 20 percent in Southern Europe to 35 percent in Scandinavia. In contrast, two child families have low proportions of zero or negative wealth, ranging from just over 20 percent in Southern and Western Europe to just over 25 percent in Scandinavia. Altogether, zero or negative wealth holdings are highest in Scandinavia, while the probability of having zero or negative wealth is lowest and the curvilinear association between the family size and the probability to own wealth is least prominent in Southern Europe. This inverse-U pattern is reflected in the distribution of family size within our sample (see Table 3 in the manuscript appendix). Over 40 percent of our sample have two children, slightly less than 20 percent have one child, and slightly less than 20 percent have three children. Roughly 10 percent are childless and 10 percent have four children or more.

**Table 1:** Proportion of observations with zero or negative wealth

	Number of children				
	0	1	2	3	4 +
Western Europe	29.69 (45.70)	23.90 (42.65)	22.24 (41.59)	23.29 (42.27)	27.32 (44.57)
Scandinavia	34.52 (47.58)	28.37 (45.09)	25.71 (43.71)	27.10 (44.45)	27.49 (44.66)
Southern Europe	23.07 (42.14)	24.62 (43.09)	23.45 (42.37)	24.05 (42.74)	25.95 (43.85)

Percentages and standard deviations in parentheses displayed. Data are weighted

**Fig. 2:**
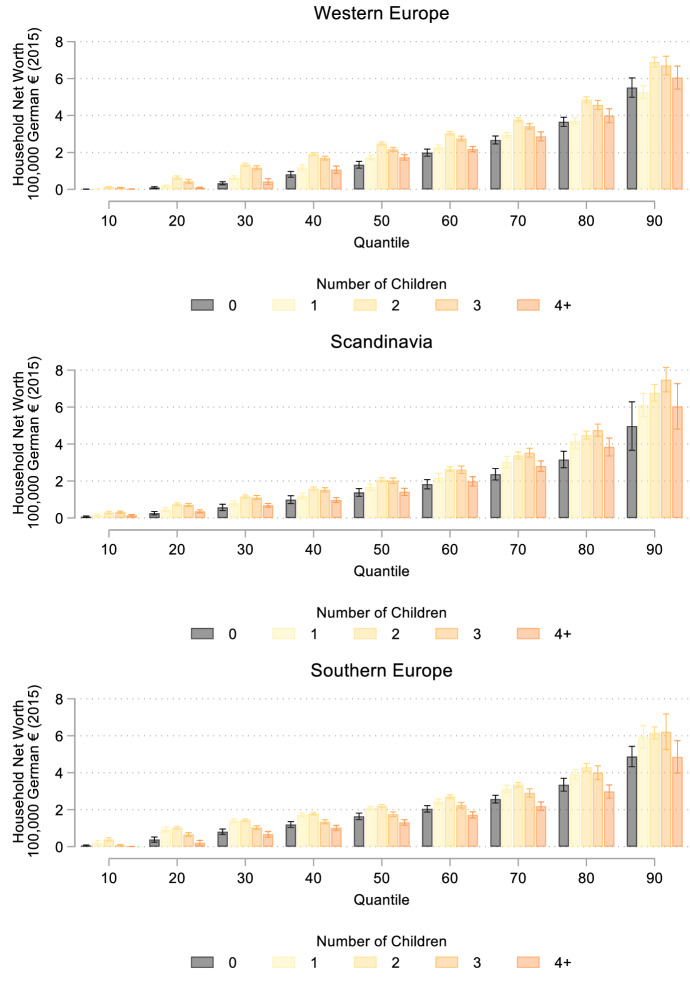
Total Household Net Worth across the Wealth Distribution by Family Size

Total household net worth by family size across the wealth distribution is displayed in Fig. 2 by geographic group. Further summary statistics by family size and wealth quantile (zero wealth, between the first and 50th quantile, and between the 50th and final quantile) are presented in Table 3 (see manuscript appendix). As can be seen in Fig. 2, family size differences in the amount of wealth owned manifest themselves across the distribution. At the 10th percentile, adults with two children own 18,500€, compared to 9500€ for adults with three children, 5500€ for one child, 2400€ for childless adults, and only 1600€ for those with four or more children. Family size differences continue to grow across the wealth distribution. At the median, men and women with two children have a net total worth of 231,400€, those with one child and three children have between 189,000€ and 203,000€, while childless individuals have an average of 145,900€ and individuals with four or more children have an average of 156,500€. At the 90th percentile, individuals with two or three children have over 100,000€ greater wealth than childless individuals and those with one or four or more children. In sum, our descriptive statistics suggest that individuals with smaller families have a wealth advantage over individuals without children or with larger families. Again, we find that welfare states are relatively similar, although the amount of wealth owned across the wealth distribution is highest in Western Europe and family size differences are least prominent in Southern Europe.

There are also other important differences by family size and wealth (see Table 3 in manuscript appendix). Marriage is more common among men and women with children compared to childless individuals and among wealthy respondents. For example, only 45 percent of childless respondents without wealth were ever married compared to 64 percent of childless wealthy individuals and nearly 99 percent of wealthy men and women with two children. Unsurprisingly, wealthier individuals are higher educated, but men and women with four or more children are less educated than individuals with smaller families regardless of their total wealth. Finally, the average number of rooms in the childhood household varies little across family size, although adults with larger family size tended to have more rooms in the family home. Wealth differences in the size of home are more considerable, with wealthier adults having grown up in larger homes.

Our index for family transfers generosity, measured in percent of average earnings, is displayed in Table 2 across birth years for each study country grouped by geographic group. As can be seen, family benefits for our birth cohorts tend to be least generous in Southern Europe, spanning between 5 percent in Spain and 10 percent in Italy, and highest in Western Europe, ranging between 10 percent in Switzerland and the Netherlands and well over 20 percent in Belgium. Our Scandinavian countries, Sweden and Denmark, hover around or just over 10 percent. In many countries, the generosity of family transfers varies very little across our cohort window of observation, for example in Austria, the Netherlands, and Ireland. The majority of countries moderately increased the generosity of their family benefits across cohorts, such as Luxembourg, Belgium, West Germany, Switzerland, Denmark, and the Southern European countries. However, a few countries cutback on family transfers across cohorts, most notably in France.

**Table 2:** The generosity of family transfers by country and birth cohort

	Birth cohort				
	1940	1945	1950	1955	1960
AUT	18.04	18.02	18.06	17.87	18.09
FRG	11.40	12.07	12.70	14.69	16.60
SWE	10.70	10.60	10.53	9.811	9.492
NLD	10.39	10.38	10.41	10.62	10.72
ESP	5.748	5.617	5.779	6.021	6.702
ITA	9.396	9.615	9.546	9.875	10.80
FRA	16.87	16.20	15.70	14.88	13.80
DNK	10.00	10.78	11.56	11.96	12.47
GRC	8.486	10.63	11.55	13.61	16.29
CHE	8.526	9.218	9.792	10.80	11.64
BEL	17.25	18.44	19.63	20.41	21.27
IRL		12.75	12.43	12.58	
LUX			21.97	23.29	24.44
PRT			7.506	7.206	8.232

Birth cohort family generosity indicator (Cohort average of annual total tax and benefit transfers for a two-parent, two-child, one-earner family expressed as the percent of average gross earnings of a production worker)

### Results from Logistic Regressions–Zero or Negative Wealth

**Fig. 3:**
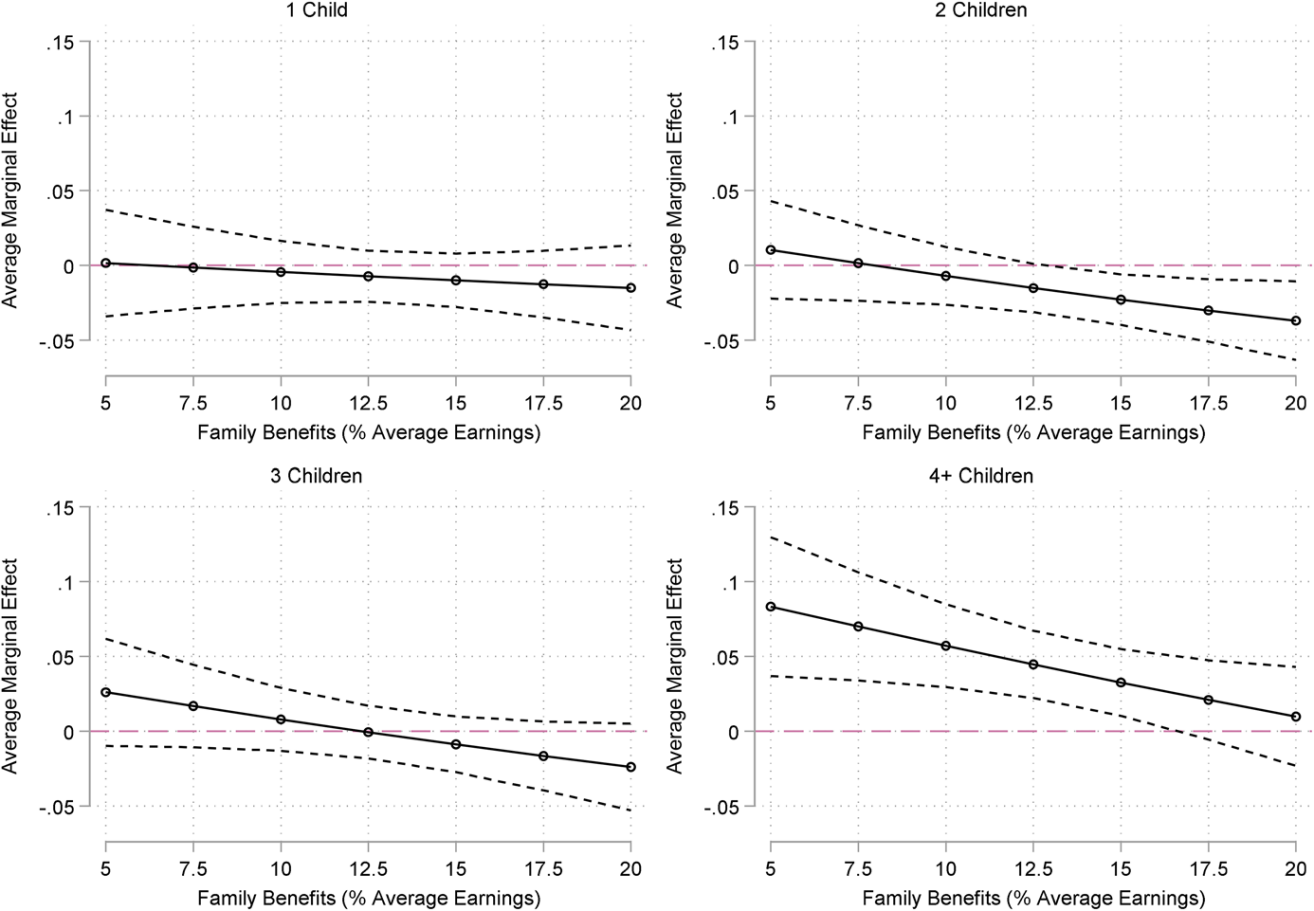
Estimated Associations between Family Size and the Probability of Zero or Negative Wealth by the Generosity of Family Transfers (ref. Childless)

The results of the logistic regressions of the probability of zero or negative wealth on family size and the generosity of family transfers are displayed in Table 4 (see manuscript appendix). Estimated associations between family size and the probability to have negative or zero wealth by the generosity of family transfers are displayed in Fig. 3.

In a model with the most basic controls—gender, age, country and birth year—we find that compared to childless adults, the odds of having zero or negative wealth are lowest for adults with 2 children (47 percent) followed by adults with one and three children (33 and 28 percent, respectively). The odds of adults with four or more children to have zero or negative wealth are considerably higher (25 percent) than childless adults. However, many family size differences become statistically insignificant after adjusting for respondents’ educational attainment, whether they had ever married, and their childhood socio-economic background. Educational attainment and ever having been married are both strongly associated with the probability of having zero or negative wealth and are known to be associated with entering parenthood and having smaller family sizes. The negative association for adults with four or more children remains large and statistically significant even after controls are introduced in the model: compared to childless adults the odds of having zero or negative wealth is 49 percent higher for adults with four or more children.

However, we find evidence that the generosity of family transfers can ameliorate the positive relationship between family size and being in debt. For families with four or more children, the probability of having negative or zero wealth is nearly 10 percentage points higher than childless adults in less generous contexts, but reach zero in the most generous contexts. In contrast, the probability of having negative or zero wealth for families with two children is roughly 5 percentage points lower than childless adults in the most generous contexts, but there are no differences in the less generous contexts.

In sum, with regard to the zero or negative wealth, our findings most closely support our hypothesis H1c: adults with two children have the lowest probability of having zero or negative wealth. However, these family size differences seem to be contingent on the generosity of family transfers, in line with Hypothesis H2c. We find that for larger families, the negative association between the probabilities of zero or negative wealth is smaller in contexts with more generous family transfers and the positive association between the probability of zero or negative wealth for adults with two children is largest in contexts with more generous family transfers.

### Results from Linear Regressions–Net Worth Percentile Rank

**Fig. 4:**
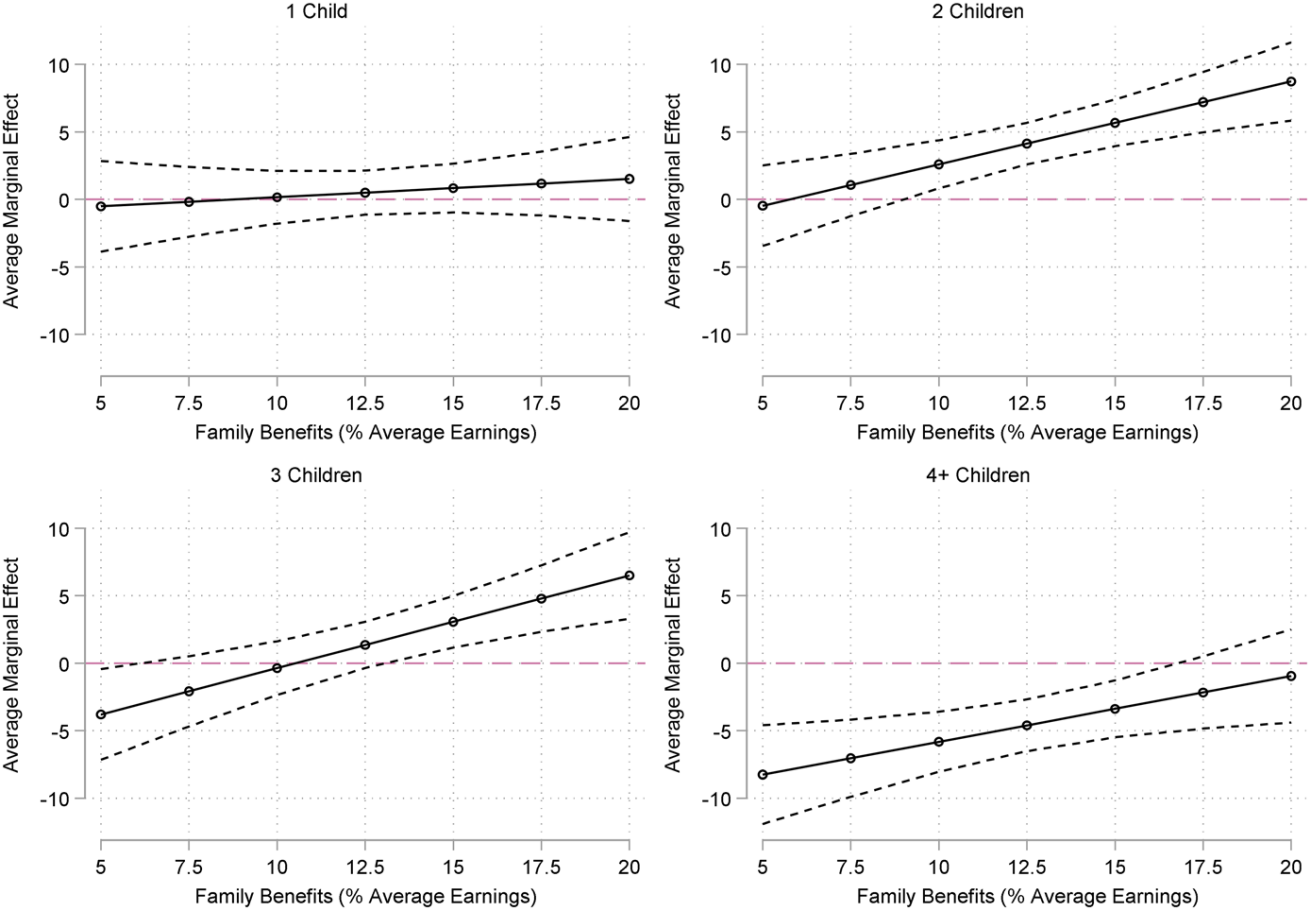
Estimated Associations between Family Size and Percentiles of Net Worth Percentile Rank by the Generosity of Family Transfers (ref. Childless)

The results of the linear regressions of net worth percentile rank on family size and the generosity of family transfers are displayed in Table 5 (see manuscript appendix). Estimated associations between family size and net worth percentile rank by the generosity of family transfers are displayed in Fig. 4.

In a model with the most basic controls, we find that adults with one to three children tend to have more wealth than childless adults. Having one and three children is associated with a 5 and 6 percentile increase in net worth rank compared to childless adults. The advantage for adults with two child is even greater: having two children is associated with a 9 percentile increase in net worth rank compared to childless adults. After adjusting for respondents’ educational attainment, whether they had ever married, and their childhood socio-economic background, the positive association between adults with two children is reduced to a roughly 4 percentile increase in net worth rank. Compared to childless adults, those with four or more children have around 4.7 percentiles lower net worth rank. Similar to the results above for zero or negative wealth, educational attainment and having ever married are both strongly associated with net worth rank and play a considerable role in attenuating the two child estimate while strengthening the four or more children estimate.

Again, we find evidence that the generosity of family transfers moderates the association between family size and wealth. Families with four or more children have nearly 10 percentiles lower net wealth rank than childless adults in contexts in the least generous contexts and continue to have less wealth in contexts with average generosity. However, this negative association becomes non-significant and reaches zero in the most generous contexts. In contrast, there are no substantial differences between adults with two or three children and adults without children in less generous contexts. However, compared to childless adults, those with two children have roughly 5 percentiles higher net worth rank in contexts with average generosity and nearly 10 percentiles higher in the most generous contexts.

In sum, our results for net worth percentile rank reflect those for zero or negative wealth, although we find larger differences between adults with three children and childless adults. Our findings most closely support hypothesis H1c: a positive relationship with wealth for adults with two or three children and a negative association for adults with large families. Again, these family size differences seem to be contingent on the generosity of family transfers in a manner postulated by H2c. We find that for larger families, the negative association with wealth is completely mitigated in the most generous contexts, while the positive association with wealth for adults with two or three children is exacerbated in contexts with more generous family transfers.

### Additional Analyses

One limitation of our analytical approach lies in macro-level confounding. Specifically, institutional or cultural differences that vary across cohorts within countries or across countries within cohorts will not be captured by our country and cohort fixed effects and may bias our estimates. Two factors are particularly worrisome: the generosity of pension schemes and housing regimes. We drew on the Comparative Welfare Entitlements Dataset (Scruggs et al., 2013) to include an indicator of pension generosity for our respondents at age 50. This indicator summarizes aspects of pension replacement rates, required years of work, the ratio of employee to employer contributions, coverage, and retirement age. In addition, we estimated the country-cohort prevalence of home ownership using SHARE. Analyses that include these indicators yield similar results and lead to the same substantive conclusions.^1^

## Discussion

In this study, we addressed two research questions: what is the association between family size, i.e. the number of children, and household wealth for adults who are preparing for or have entered retirement, and does the generosity of family transfers, i.e. the extent that countries compensate families for the costs of children, moderate the association between family size and wealth? We hypothesized that the number of children will be associated with less wealth (H1a), possibly because the costs of children exceed household needs and parents’ ability to save. In contrast, the number of children will be associated with higher wealth (H1b), especially if couples save more to prepare for the costs of childrearing and bequest motives then. Alternatively, the cost of a first child together with fulfilling the norms associated with a two child family might lead to a nonlinear association between family size and wealth where one child families and families with many children are disadvantaged relative to two child families (H1c). Further, we argued that if family transfers reduces the monetary costs of children, then either the negative association between wealth and family size is smaller in contexts with more generous family transfers (H2a) or the positive association between wealth and family size is larger in contexts with more generous family transfers (H2b). In addition, we speculated that the negative association between one child families and families with many children and wealth is smaller in contexts with more generous family transfers, while the positive association between two children and wealth is larger in contexts with more generous family transfers (H2c).

We used data from the Survey of Health, Ageing, and Retirement in Europe (SHARE) to estimate the relationship between family size and wealth of men and women between ages 50–65, born 1939–1967 from 14 European countries, and Gauthier's (2011) comparative family policy dataset to estimate whether the generosity of family transfers moderates the association between family size and wealth. Results from logistic regressions most closely support hypotheses H1c and H2c: larger family sizes are more likely to have zero or negative wealth, but that the generosity of family transfers can ameliorate those negative associations. In contrast, two child families are less likely to have zero or negative wealth, but only in the most generous of contexts. These findings were reflected in our analyses on net worth percentile rank, which demonstrated a positive relationship with wealth for two and three child families in all but the least generous contexts, but a negative relationship with wealth for two and three child families except for in the most generous settings.

This study contributes both theoretically and empirically to the literature on wealth accumulation and inequality. Theoretically, we extend the life-cycle models of wealth accumulation found in both the sociological and economic literatures. Partially due to the lack of comparative research on wealth, there has been little thought on how context might interact with the association between family size and wealth. We argue that it is integral to account for the generosity of family transfers when hypothesizing about how the number of children will influence wealth accumulation and the amount of wealth that adults own. Our study is one of the first accounts of family size differences in wealth ownership in a sample of European countries that assess the role that family transfers can play.

Indeed, we show that context, and specifically the generosity of family transfers, matter in important ways. Many of the family size differences we found were only evident in contexts with low or high transfer generosity. For example, our results are in line with the argument that couples with two or three children save more to prepare for the costs of childrearing and bequest motives, but may be only able to do so once costs associated with children are covered by state transfers. Moreover, our findings support the idea that the costs of having many children exceed household needs and parents’ ability to save, driving some households into debt. However, the contexts with generous family transfers seem to be able to keep large families from falling into debt, but not enable them to accumulate wealth as adults with fewer children could.

Empirically, our study sought to provide a rich description of the relationship between family size, the generosity of family transfers, and wealth among adults, but was not able to untangle the mechanisms linking these factors. One possibility might lie differential marital status and household division of labour by family size. Women in households with one or two children may be more likely to quickly re-enter and remain active in the labour market than women with three or more children. Therefore, dual-earner households with one or two children would have more to spend or save than single-earner households with three or more children. Alternatively, couples with three or more children may expect that their children will support them in old-age and offset any wealth disadvantage. The intergenerational transmission of fertility behaviour might also play a role. Women from large families are more likely to have larger families themselves (Fasang, 2016), and adults with many siblings inherit less wealth than adults with fewer siblings (Lersch, 2019). Future research should investigate avenues for causal research and to shed light on these possible mechanisms. We see at least two possibilities. First, scholars could use harmonized longitudinal data to perform a small-N country comparison of how parity transitions affect wealth accumulation over the life course. Second, scholars could identify dramatic temporal or regional variation in family transfers that might act as a “natural experiment” to identify the effects of family transfers on wealth accumulation across adults with smaller and larger family sizes. In addition, future research should attempt to estimate quantile treatment effects to estimate how family size differences vary across the wealth distribution (e.g. Maroto, 2018).

One of our main contributions was to demonstrate that whether the number of children is associated with wealth may depend on two factors that have been overlooked in past research. Moreover, these two factors may account for some of the diverging findings. First, the association between family size and wealth varies by the number of children. We find no differences between childless adults and those with one child, a positive relationship between two and three child families, and a negative relationship for large families. It is possible that some null results may be attributable to measuring family size continuously (Ozawa & Lee, 2006; Painter et al., 2015; Tamborini & Purcell, 2016). Second, we provide evidence that the generosity of family transfers can ameliorate the negative association between larger family sizes and the probability of wealth ownership, but exacerbate the association between small families and the amount of wealth owned. Studies conducted with US data should take state variation in taxation, e.g. state-level earned income tax credits, into account when estimating the association between family size and wealth.

It is important to gain a better understanding of how family transfers moderates the relationship between family size and wealth accumulation. We provided evidence that countries can limit the negative impact that large family sizes have on wealth accumulation by compensating for the direct costs of children. However, we also showed that generous family benefits families with two children the most. While the nonlinear association between family size and wealth will most likely translate into a non-significant near-zero linear association, our results indicate that this association may turn positive in the most generous contexts because of the advantaged position of two and three child families. Indeed, Semyonov and Lewin-Epstein (2013) report positive associations between household size and wealth in Belgium, Switzerland, and Denmark; three countries with relatively high and increasing family transfers across our observation period. In the context of decreasing fertility and family sizes across Europe, the role of family transfers in particular for two child families could have important implications for wealth inequality among adults as they prepare to enter retirement.

## Data Availability

The data that support the findings of this study are available for scientific use at http://www.share-project.org.

## Appendix

See Tables

**Table 3:** Sample summary statistics

Number of Children	0	0	0	1	1	1	2	2	2	3	3	3	4 +	4 +	4 +
Percentile	0	0–50	50–100	0	0–50	50–100	0	0–50	50–100	0	0–50	50–100	0	0–50	50–100
Household Net Worth	−0.03	0.4	7.76	−0.03	0.42	6.57	−0.05	0.49	7.47	−0.04	0.46	9.38	−0.04	0.37	8.31
Family Transfers	13.27	11.97	11.63	12.92	12.57	11.94	12.32	11.96	11.92	12.67	11.99	12.33	12.97	12.17	12.49
*Marital Status*															
Never Married	0.55	0.55	0.36	0.1	0.07	0.03	0.03	0.02	0.01	0.01	0.02	0.01	0.07	0.05	0.01
Married	0.45	0.45	0.64	0.9	0.93	0.97	0.97	0.98	0.99	0.99	0.98	0.99	0.93	0.95	0.99
Years of Education	12.02	10.91	12.1	12.01	11.14	11.84	11.76	10.47	11.76	11.66	9.96	11.72	10.06	8.44	10.72
Number of Rooms	4.58	5.14	5.23	4.38	4.79	4.95	4.42	4.61	4.83	4.44	4.58	4.88	4.64	4.95	5.05
*Gender*															
Male	0.61	0.61	0.52	0.49	0.48	0.48	0.5	0.47	0.51	0.52	0.46	0.5	0.51	0.48	0.48
Female	0.39	0.39	0.48	0.51	0.52	0.52	0.5	0.53	0.49	0.48	0.54	0.5	0.49	0.52	0.52
Age	58.1	57.41	57.66	58.29	57.78	57.92	58.35	57.81	58	58.29	57.97	58.16	58.29	58.11	58.74
Year of birth	1957	1953	1952	1958	1953	1952	1958	1953	1952	1957	1952	1952	1956	1952	1951
*N*	1,554	2,262	3,212	2,375	3,224	6,499	4,976	6,331	17,270	2,411	3,325	7,576	1,515	2,360	3,639

^3^,

**Table 4:** Logistic regression results of negative and positive wealth on family size and the generosity of family transfers

	1	2	3
*Number of Children (ref.: 0)*			
1	0.751*** (0.05)	0.938 (0.08)	1.072 (0.26)
2	0.682*** (0.04)	0.869 + (0.07)	1.286 (0.28)
3	0.789*** (0.06)	0.994 (0.08)	1.474 (0.36)
4 +	1.253* (0.12)	1.490*** (0.15)	2.522** (0.75)
*Gender (ref.: male)*			
Female	0.963 (0.03)	0.957 (0.03)	0.958 (0.03)
Age	59.902*** (22.69)	60.944*** (23.19)	61.069*** (23.25)
Age^2^	0.971*** (0.00)	0.971*** (0.00)	0.971*** (0.00)
*Marital Status (ref.: never married)*			
Married		0.591*** (0.06)	0.593*** (0.06)
Number of Rooms		0.999 (0.01)	1.000 (0.01)
Years of Education		0.930*** (0.02)	0.930*** (0.02)
Years of Education^2^		1.002** (0.00)	1.002** (0.00)
Family Transfers			0.960 (0.02)
*Number of Children*^***^*Family Transfers (ref.: 0)*			
1			0.989 (0.02)
2			0.969* (0.02)
3			0.969 + (0.02)
4 +			0.959* (0.02)
Constant	0.000*** (0.00)	0.000*** (0.00)	0.000*** (0.00)
Pseudo-R^2^	0.386	0.388	0.389
N	73,228	73,228	73,228

Exponentiated coefficients; Robust clustered standard errors in parentheses; Sig.: + *p* < 0.1, **p* < 0.05, ***p* < 0.01, ****p* < 0.001; Country and birth year fixed effects omitted from table; Continuous variables not centred; Data weighted

4,

**Table 5:** Linear regression results of net worth percentile rank on family size and the generosity of family transfers

	1	2	3
*Number of Children (ref.: 0)*			
1	5.387*** (0.79)	0.407 (0.84)	−1.195 (2.59)
2	9.280*** (0.72)	3.934*** (0.80)	−3.537 (2.29)
3	6.066*** (0.80)	1.192 (0.89)	−7.236** (2.58)
4 +	−1.619 + (0.94)	−4.707*** (0.99)	−10.693*** (2.79)
Gender (ref.: male)			
Female	0.039 (0.41)	0.568 (0.40)	0.552 (0.39)
Age	−27.274*** (1.43)	−26.895*** (1.41)	−26.952*** (1.41)
Age^2^	0.210*** (0.01)	0.206*** (0.01)	0.207*** (0.01)
*Marital Status (ref.: never married)*			
Married		11.615*** (0.92)	11.653*** (0.91)
Number of Rooms		0.067 (0.08)	0.060 (0.08)
Years of Education		1.414*** (0.16)	1.411*** (0.16)
Years of Education^2^		−0.013 + (0.01)	−0.013 + (0.01)
Family Transfers			−0.078 (0.24)
*Number of Children*^*^*Family Transfers (ref.: 0)*			
1			0.136 (0.19)
2			0.614*** (0.17)
3			0.687*** (0.19)
4 +			0.487* (0.20)
Constant	941.137*** (41.50)	913.981*** (40.86)	914.815*** (40.89)
Pseudo-R^2^	0.263	0.288	0.289
N	73,228	73,228	73,228

Unstandardized odds-ratios; Robust pooled standard errors in parentheses; Sig.: + *p* < 0.1, **p* < 0.05, ***p* < 0.01, ****p* < 0.001; Country and birth year fixed effects omitted from table; Continuous variables not centred; Data weighted

5,

**Table 6:** Comparison of person and country clustered standard errors

	Zero or Negative Wealth		Net Worth Percentile Rank	
	Persons	Countries	Persons	Countries
*Number of Children (ref.: 0)*				
1	1.072 (0.260)	1.072 (0.110)	−1.195 (2.587)	−1.195 (1.474)
2	1.286 (0.285)	1.286* (0.158)	−3.537 (2.293)	−3.537* (1.359)
3	1.474 (0.358)	1.474** (0.181)	−7.236** (2.584)	−7.236*** (1.429)
4 +	2.522** (0.748)	2.522 + (1.225)	−10.693*** (2.789)	−10.693** (3.065)
*Gender (ref.: male)*				
Female	0.958 (0.034)	0.958 (0.043)	0.552 (0.394)	0.552 (0.674)
Age	61.069*** (23.254)	61.069*** (22.620)	−26.952*** (1.410)	−26.952*** (3.137)
Age^2^	0.971*** (0.003)	0.971*** (0.003)	0.207*** (0.012)	0.207*** (0.027)
*Marital Status (ref.: never married)*				
Married	0.593*** (0.057)	0.593*** (0.068)	11.653*** (0.911)	11.653*** (0.932)
Number of Rooms	1.000 (0.011)	1.000 (0.026)	0.060 (0.082)	0.060 (0.194)
Years of Education	0.930*** (0.016)	0.930*** (0.019)	1.411*** (0.160)	1.411** (0.434)
Years of Education^2^	1.002** (0.001)	1.002** (0.001)	−0.013 + (0.007)	−0.013 (0.017)
Family Transfers	0.960 (0.024)	0.960** (0.014)	−0.078 (0.237)	−0.078 (0.249)
*Number of Children*^*^				
Family Transfers (ref.: 0)				
1	0.989 (0.017)	0.989* (0.006)	0.136 (0.190)	0.136 (0.117)
2	0.969* (0.015)	0.969*** (0.007)	0.614*** (0.170)	0.614*** (0.111)
3	0.969 + (0.017)	0.969*** (0.007)	0.687*** (0.191)	0.687*** (0.117)
4 +	0.959* (0.020)	0.959 (0.027)	0.487* (0.204)	0.487* (0.189)
Pseudo-R^2^	0.389	0.389	0.289	0.289
N	73,228	73,228	73,228	73,228

Unstandardized coefficients; Robust pooled standard errors in parentheses; Sig.: + *p* < 0.1, **p* < 0.05, ***p* < 0.01, ****p* < 0.001; Country and birth year fixed effects, and constant omitted from table; Continuous variables not centred; Data weighted

6,

**Table 7:** Comparison of the socio-demographic composition of all cases without exclusions, with missings on wealth, with missings on other variables, and the analysis sample

	Full Sample		Missing on Wealth	Other Missing	Analysis Sample
	Average	*N*			
Household Net Worth	3.954	75,820		3.673	3.962
Number of Children	1.911	103,127	1.826		1.94
Family Transfers	12.263	104,735	12.339	12.452	12.226
Marital Status		103,747			
Never Married	0.094		0.123		0.08
Married	0.906		0.877		0.92
Years of Education	11.547	103,563	12.171		11.329
Number of Rooms	4.86	104,735	5.166		4.752
Gender		104,735			
Male	0.494		0.468	0.514	0.502
Female	0.506		0.532	0.486	0.498
Age	57.939	104,735	57.634	58.130	58.039
Year of birth	1953	104,735	1953	1953	1954
*N*			28,915	3,082	73,228

^7^

## Footnotes

1 Note that we do not include these analyses, because the Comparative Welfare Entitlements Dataset does not include information on Luxembourg. Moreover, the estimate for home ownership is highly statistically insignificant and does not contribute to the regression in terms of model fit or attenuation of any relevant coefficients.

## Code Availability

Replication files will be made available at https://www.zachary-vanwinkle.com/file-share.

## Declarations

### Human and Animal Participants

This article does not contain any studies with human participants or animals performed by the authors.
